# A prospective randomized pilot study evaluating the scar outcome after gluteal dermis fat graft with and without kinesiotaping

**DOI:** 10.1007/s10792-022-02304-7

**Published:** 2022-04-02

**Authors:** Annemarie Klingenstein, Aylin Garip-Kuebler, Daniel R. Muth, Christoph Hintschich

**Affiliations:** Department of Ophthalmology, Ludwig-Maximilians-University, Klinikum der Universität München, Campus Innenstadt Mathildenstraße 8, 80336 Munich, Germany

**Keywords:** Dermis fat graft, Kinesiotaping, Scar therapy, Enucleation

## Abstract

**Purpose:**

To compare gluteal wound healing after dermis fat graft (DFG) implantation in patients with and without local application of kinesiotapes.

**Methods:**

In this prospective, single-center analysis, 16 patients who underwent DFG implantation were randomized in two groups. Wound healing was compared 4–6 weeks after therapy and 3 months later (after application of 2 cycles of kinesiotaping for 2–3 weeks in the case and no specific therapy in the control group). Demographic data, patient content and wound healing were assessed. Scarring was graded (0–3) by evaluation of photodocumentation by 2 blinded, independent observers.

**Results:**

Mean scar grading by both observers decreased from 2.31 ± 0.48 to 1.13 ± 0.72 in the case and from 2.38 ± 0.52 to 1.44 ± 0.50 in the control group with interobserver agreement on scar grading being substantial to almost perfect in both groups. Scar length decreased significantly in both groups (*p* = 0.008). Scar prominence decreased in 2/3 of cases in the case and 1/3 in the control group. Scar coloring significantly improved in the case group alone (*p* = 0.031).

**Conclusion:**

No functionally impairing or painful scar developed. No adverse effects occurred after kinesiotaping. Gluteal scars shortened significantly over time and were significantly paler in the case group. Kinesiotaping may improve scar elevation over no specific scar therapy.

## Introduction

Patients who require enucleation should be supplied with an orbital implant in order to obtain the best possible functional and cosmetic result. Dermis fat graft (DFG) implantation has been an established surgical procedure for decades [1] and excellent aesthetical and functional results regarding the socket are reported [2, 3] with low complication rates (e.g., delayed healing at the site of explantation in 3.6% of cases) [4].

An alternative procedure is enucleation and implantation of anorganic material with both porous and nonporous implants generally being well tolerated, and complication rates being low [5, 6]. Yet, long-term exposure of the implant is possible and rates may reach 24.7%, 23.5% and 76.5% for hydroxyapatite, bioceramic and Medpor^®^, respectively [7, 8]. Our long-term clinical experience favors the results of autologous fat for implantation: We note a very low rate of conjunctival extrusion of the implant and of postenucleation syndrome as well as extremely rare rejection reactions due to the autologous implant material.

Yet, in comparison to alloplastic implants, the use of DFG results in possibly functionally and cosmetically restrictive scarring at the (gluteal or abdominal) donor site. In our clinic, we routinely perform DFG harvesting from the gluteal region at 5 cm above the middle of a connecting line between the anterior superior iliac spine and the ischial tuberosity [9].

In scarring, myofibroblasts produce collagen during healing. This process can be elevated and result in hypertrophic scarring or even keloid formation as a fibroproliferative disorder [10], resulting from a patient’s skin type, healing tendencies or predispositions. In order to minimize post-surgical scar development, correct incision planning, skin closure, and postoperative care must be considered essential [11]: Primary surgical wound closure as the second step of the reconstructive ladder is indicated in significant tissue deficit precluding nonsurgical management [12]. For best possible healing after DFG harvesting, the circular tissue defect is closed in two layers following the principles of surgical wound closure: Buried subcutaneous sutures are advocated whenever possible to reduce the tension on skin sutures, close dead space beneath a wound, and allow for early suture removal [13]. Furthermore, interrupted horizontal mattress sutures applied, as one of the most commonly used skin closure methods, promote wound edge eversion and induce less scarring postoperatively [14, 15]. They allow for skin edges to be brought together over a distance [15]. Additionally, for best possible healing, the sutures are adapted in conformity to the Langer lines parallel to the natural orientation of collagen fibers in the dermis.

Different treatment algorithms for prophylaxis and therapy of hypertrophic scars or keloids are possible, but there is no established treatment strategy [16]. Early treatment possibilities include topical treatment with silicone (as sheets, gels, sprays or foams) [17, 18] as first-line therapy which decrease the scar size [19, 20], onion extract creams or oils as well as physical pressure or massaging with or without bandaging or taping to apply pressure [16].

Kinesiotaping is supposed to weaken subcutaneous adhesions and thus improve the appearance and softness of the scar. Physical therapists apply kinesiotapes tensionless over the complete length with the base in the center of the scar, oftentimes treating scars over joints. Up to now, only few studies have investigated the beneficial use of tapes to improve postoperative scarring [21, 22].

Hypertrophic scars that do not improve by 6 months should be managed intensively with intralesional steroid injections or alternate modalities [17]. Intralesional steroid injections reducing collagen production and thus increasing degradation within the fibrous portion of the scar are reportedly highly effective in the management of hypertrophic scars [23, 24]. Furthermore, laser therapy may flatten elevated scars and lighten pigmentation [25, 26] and cryotherapy and repeated surgery have been reported [16, 27].

In this prospective, randomized, mono-centric, single-center analysis, we compared wound healing after gluteal DFG harvesting: Patients in the case group were treated by applying kinesiotape after initial evaluation of the scar 4–6 weeks after therapy, whereas patients in the control group did not apply any treatment to the resulting scar. We hyposthesized that gluteal scarring can be improved functionally as well as cosmetically by kinesiotaping of the scar and hypertrophic scarring or keloid formation can thus be prevented.

## Methods

The ethics committee of Ludwig-Maximilians-University, Munich/Germany approved the prospective, randomized, case–control evaluation of the gluteal wound healing process of patients having undergone enucleation and primary DFG implantation as performed in this trial (vote number 19-093, registration date 10/04/2019) at the oculoplastic department of the eye clinic of Ludwig-Maximilians-University, Munich/Germany after evaluation and approval of the study protocol. The study is in accordance with the Declaration of Helsinki. Written informed consent was obtained from all patients prior to inclusion into the study. The study is registered in the Deutsches Register Klinischer Studien (www.drks.de; number DRKS00023111, registration date 05/10/2020).

Patient eligibility criteria included patients having undergone DFG harvesting over 18 years of age. Exclusion criteria were skin alterations as well as known allergic reactions to tape.

The primary endpoint of the study was evaluation of the gluteal scar (length, color, prominence, indentation, visibility) 4–6 weeks post-surgery as well as 3 months later. The secondary endpoint was patient content regarding functionality and cosmesis.

Sample size was calculated as per the expected number of surgeries over a period of two years. Originally, inclusion of 50 patients was planned (5 sets of 10 numbers evenly randomized to the case and control groups by the Research Randomizer software (https://www.randomizer.org/)). Numbers were sealed in sequentially numbered envelopes in order to conceal the sequence until interventions were assigned.

The patients in the case group locally applied kinesiotapes (hypo-allergenic spiral tapes from Atex Medical, CE certification as a medical product by Medical Device Directive 93/42/EEC since 2007/47/EC, Standards ISO 10993-1, ISO 14971) starting after a first evaluation and documentation of the scar 4–6 weeks after surgery for 2 cycles of 2–3 weeks. These skin-colored tapes do not penetrate the epidermis, do not contain transdermal medication and function mechanically. Application of the tape is simple and approved for doctors as well as for physical therapists.

### Dermis fat graft harvesting

Surgery was performed under general anesthesia employing the technique described by Smith et al. [1]. In brief, the DFG with a standard size of 25 mm in diameter and thickness in adults was harvested from the gluteal region of the patients incising the epidermis superficially with a No. 15 scalpel, injection of saline intradermally at the donor site, then dissecting and separating the epidermis from the dermis layer by a No. 20 blade. Then, after deep transection of the fat layer, the DFG was explanted and the wound closed in two layers with three 2.0 absorbable subcutaneous sutures (Vicryl^®^, Ethicon, Johnson and Johnson Medical GmbH, Norderstedt, Germany in both groups) after control of obvious bleeding using bipolar cautery. Closure of the skin was performed with 2.0 silk mattress sutures. Excision of “dog’s ears” for better-wound adaptation, if performed, was annotated for analysis.

At the donor site, steristrips were placed perpendicularly over the sutured wound followed by a sterile dressing for 2 days. Wound adaptation was then checked. Gluteal sutures were removed at 10–12 days after surgery.

### Application of kinesiotapes

In the case group, the kinesiotapes were fitted over the clean scar after having checked and documented proper wound healing thus far. As the resulting gluteal scar is approximately 4 cm in length, we applied kinesiotapes in size “small” (Atex Medical 28 × 36 mm, approximate diagonal 46 mm). Depending on the length of the individual scar, one to maximally two tapes was/were applied diagonally over the full length of the scar covering the scar completely. First, the tissue was put slightly on stretch by the patient standing upright with the thigh slightly bent. Second, the tape was molded to the skin with no to minimal stretch. Then, gentle rubbing of the area was performed for a couple of seconds to heat activate the glue of the tape.

The tapes were then left in place for 2–3 weeks until they could be peeled off easily. Another cycle of kinesiotaping was applied if the scar was free of irritation (all cases). No further topical treatment was applied to the scar.

### Postoperative evaluation and assessment of the gluteal scar

Postoperative follow-up visits were performed 4–6 weeks after surgery prior to first prescription of the prosthesis (V1), as well as another 3 months later (V2). At both points in time, overall patient content was elaborated (binary response noted, yes or no, respectively).

**Fig. 1:**
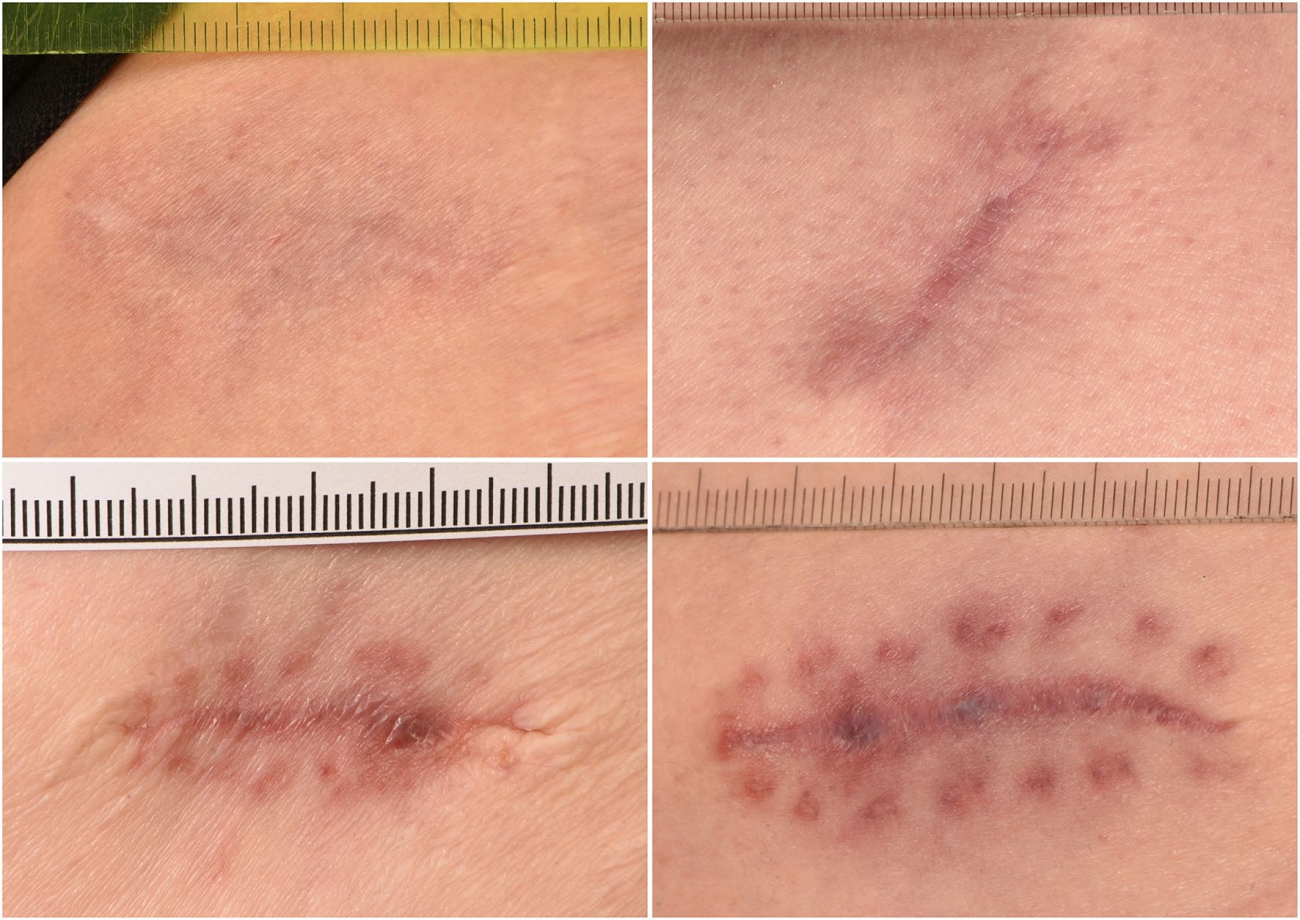
Scar graded as not visible (grade 0, upper left), minimally visible (grade 1, upper right), moderately visible (grade 2, lower left) and maximally visible (grade 3, lower right)

Wound healing was evaluated within the orbit and at the gluteal donor site of the DFG. Gluteal wound healing was documented by a still photographed close-up of the donor site at approximately 30 cm distance using a digital camera with a metric scale (calibration in cm and mm) placed carefully next to the scar without covering any parts. The photographs have been taken with a Nikon D7200 camera (Tokyo, Japan), Nikkor AF-S Micro 105 mm 1:2, 8 G ED lens, Hensel flash system (Würzburg, Germany) by our clinic photographer. The pictured gluteal section measured approximately 10 × 10 cm and excluded the perianal region. Cosmetic evaluation of the scar was performed by two blinded observers separately using the same lighting and computer screen. The visibility of the scar was graded from grade 0 (scar not visible), 1 (minimally visible), 2 (moderately visible) and 3 (maximally visible) at both examinations. For better visual reference regarding scar grading, please see Fig. 1. Additionally, total length at V1 and V2 as well as final color (erythema or normal skin color), prominence and indentation (presence or absence, respectively) of the scar was assessed.

Potential wound-healing confounders included patient gender (female or male), patient age at surgery, surgical resection of so-called “dog’s ears” for better-wound adaptation, patient body mass index (BMI), the surgeon (1, 2, and 3) and anticoagulation.

### Statistical analysis

Demographic data, as well as comparison of scar grading were analyzed by Fisher’s exact test. The changes of the scar from baseline (4–6 weeks after surgery) to next follow-up (3 months later) were analyzed by Wilcoxon-signed rank test. Interobserver reliability was compared by Kappa test. For all tests, *p* < *0.05* was considered to be statistically significant.

## Results

Analysis was completed in all 16 patients, including 8 in both the case group (3 female), and 8 in the control group (4 female, *no statistically significant difference, n.s.*). Enucleation and primary implantation of an autologous DFG for this pilot study were performed between 04/2019 and 10/2020 (study inclusion at V1 4–6 weeks post-surgery, 18 months recruitment period). In this interim analysis, we included 16 patients that were evenly randomized to the case and control group (1 set of 10 patients, 6 patients allocated from set 2) due to the lower number of operations performed during the global pandemic than originally assumed in the study protocol. Respective mean, median, and range of age at surgery were 52.7, 55.7, and 24.7–69.7 years for the case, and 58.0, 60.8, and 24.0–85.0 years for the control group (*n.s.*). As the DFG is an autologous implant, a rejection reaction is rare and has not been reported within this patient collective.

V1 took place 44 ± 16 days post-surgery in the case and 37 ± 12 days in the control group (*n.s.*). V2 was at 131 ± 31 days post-surgery in the case and 125 ± 16 days in the control group (*n.s.*). Due to COVID-related quarantine, one patient of the case and one patient of the control group had to postpone their originally scheduled V2 and V1, respectively. Patient height was 173.8 ± 9.0 cm in the case and 172.3 ± 13.0 cm in the control group (range 165-184 cm and 154-189 cm, respectively, *n.s.*). Patient weight was 83.3 ± 11.7 kg in the case and 83.8 ± 27.4 kg in the control group (range 70–97 kg and 53–130 kg, respectively, *n.s.*). This resulted in a BMI of 27.1 ± 3.3 kg/m^2^ in the case and 28.3 ± 8.9 kg/m^2^ in the control group (range 22.0–32.0 kg/m^2^ and 20.0–48.0 kg/m^2^, respectively, *n.s.*).

No patient had previous history of hypertrophic scar formation. One patient of the control group was operated under systemic anticoagulation (100 mg aspirin daily). Group comparison of the surgeon performing the operation (1–3) and performing wound closure with so-called “dog’s ears” (in 4 patients per group) to improve wound adaptation was not significant, respectively.

Enucleation with DFG harvesting was performed due to painful amaurosis in 8, uveal melanoma in 6 (recurrence in 3 and secondary glaucoma in 3), buphthalmos in 1 and posttraumatic perforation of the globe in 1 case(s). No patient presented with postoperative complications as infection, bleeding or reported any functional motility restriction resulting from the scar at V1 or V2 (yes or no answer modality). In the case group, no skin alterations resulting from kinesiotaping were observed.

Mean scar grading at V1 and V2 by observers 1 and 2, total scar length in cm, noted color, scar prominence or indentation (yes or no, respectively) and visibility grading at V1 and V2 are shown in Table 1.

**Table 1:** Scar parameters and grading. ± SD, 95% confidence interval in parenthesis

Scar parameters	Case group V1	V2	Control group V1	V2
Mean scar length [cm]	4.16	3.79*	4.10	3.88*
Prominence	37.5%	12.5%	37.5%	25.0%
Color	100%	37.5%*	87.5%	62.5%
Mean grading 0–3				
Observer 1	2.38 ± 0.52 (1.94–2.81)	1.13 ± 0.64 (0.59–1.66)	2.38 ± 0.52 (1.94–2.81)	1.38 ± 0.74 (0.75–2.00)
Observer 2	2.25 ± 0.46 (1.86–2.64)	1.13 ± 0.83 (0.43–1.82)	2.38 ± 0.52 (1.94–2.81)	1.50 ± 0.76 (0.87–2.13)
Mean Obs. 1 + 2	2.31 ± 0.48	1.13 ± 0.72	2.38 ± 0.52	1.44 ± 0.50

*p < *0.05* Wilcoxon signed-rank test

**Fig. 2:**
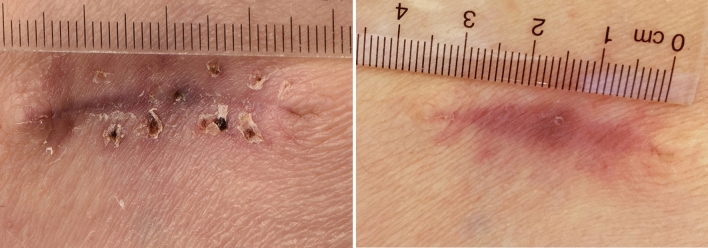
Gluteal scar without specific therapy. Scar graded as 3/3 (observer 1/2) at **a** V1 (26 days after surgery; 3.5 cm) and as 2/2 at **b** V2 (117 days after surgery; 3.4 cm) without kinesiotaping

**Fig. 3:**
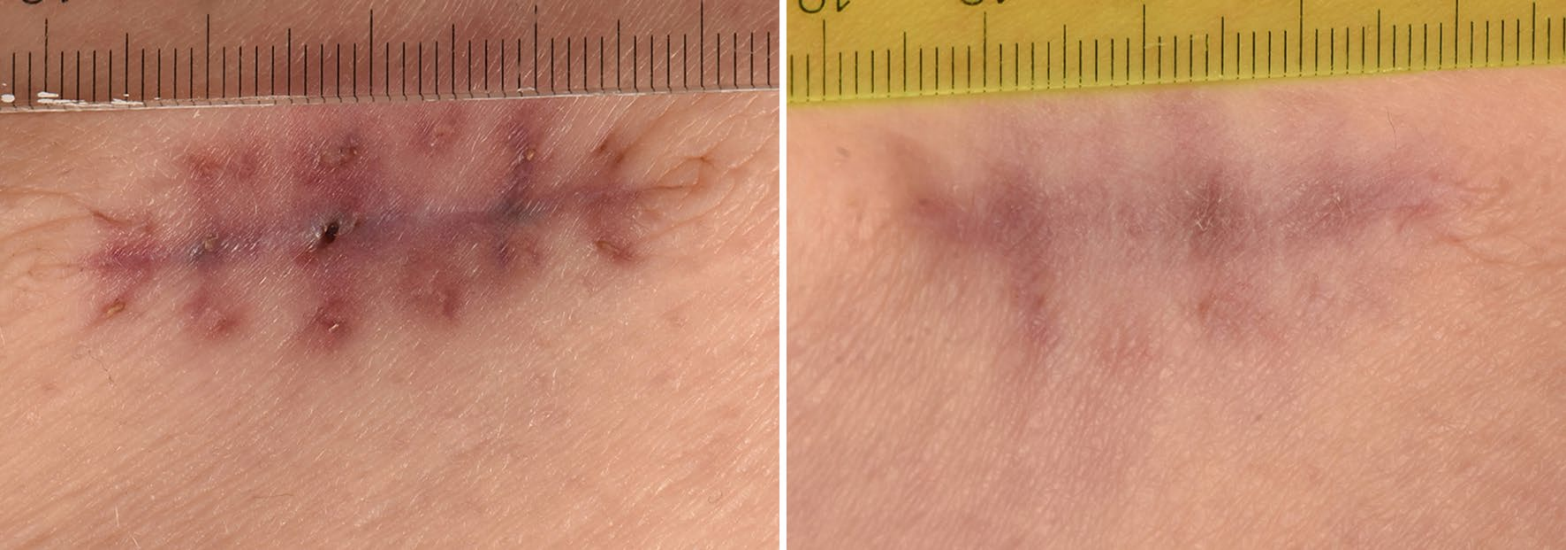
Gluteal scar treated with kinesiotapes. Scar graded as 3/3 at **a** V1 (also 33 days after surgery; length 3.9 cm) and graded as 1/2 at **b** V2 (124 days after surgery; length 3.6 cm) after application of 2 cycles of kinesiotaping demonstrating less scar coloring at V2

Mean scar grading by the 2 observers decreased from 2.31 ± 0.48 (V1) to 1.13 ± 0.72 (V2) in the case and from 2.38 ± 0.52 (V1) to 1.44 ± 0.50 (V2) in the control group. Grading decreased more in the case group, yet this was not significant (0.504; Fisher’s exact test). Scar grading improved from V1 to V2 in 8/8 cases (observer 1) and 7/8 cases (observer 2) in the case and in 6/8 cases graded (both observers) in the control group. This was statistically significant (observer 1 *p* = 0.008 and 2 *p* = 0.016 case group and observer 1 and 2 *p* = 0.031 control group, respectively; Wilcoxon signed-rank test). Interobserver agreement evaluated by Kappa test was 0.714 at V1 for the case and 1.000 for the control group and 0.610 and 0.784 at V2 for the case and control group, respectively (with strength of agreement of Cohens Kappa being substantial at 0.61–0.80 and almost perfect at 0.81–1.00). [28] Scar length decreased significantly in both groups over time from V1 to V2 (*p *= 0.008 (mean change 0.38 cm, 9.4%) case group, *p* = 0.008 (mean change 0.23 cm, 6.0%) control group, Wilcoxon signed-rank test, respectively). Scar prominence decreased in two out of three cases in the case and in one out of three cases in the control group from V1 to V2. Scar color decreased significantly from V1 to V2 in the case group (*p* = 0.031), but not in the control group (*p* = 0.500; Wilcoxon signed-rank test, respectively). Figures 2a, b and 3a, b give an example of scar devolution with and without kinesiotaping.

No patient of either the case or control group reported dissatisfaction with the functional or esthetic result of the scar at V1 or V2.

## Discussion

Although DFG harvesting is a routine surgical intervention, we could not find any data on prospective evaluation of the resulting scar at the donor site in common medical databases. In our opinion, such data are of great interest, as patients’ expectations are continuously growing. Thus, focus on function and cosmesis is mandatory.

The most important results of this study include that apparently, kinesiotaping of the gluteal scar after DFG harvesting leads to significant decrease in scar color at 3 months after the beginning of taping compared to the control group. Additionally, the scars significantly shortened after this time interval in both patient groups. Scar prominence improved in 2/3 of cases in the case and 1/3 of patients in the control group. Scar grading improved gradually in most patients of both groups (with interobserver variability being substantial to almost perfect).

Hypertrophic scars must still be considered a challenge in wound rehabilitation with different treatment options available. Treatment should be based on scar location, quality, size as well as patient-specific factors as preferences, and expectations [29]. Besides cosmetical problems, functional impairment such as contractures and subjective symptoms as pruritus may significantly affect the patients' quality of life [10].

Following the recent understanding, wound healing includes the inflammatory (2–3 days), the proliferative (3–6 weeks) and the remodeling phase (> 1 year) which partly overlap [10, 17]. Scar management is intimately connected to these stages [17]. The remodeling phase is at its peak within the first three months after surgery in which incisions destined to develop hypertrophic response will begin to reveal themselves [17]. Once hypertrophic scarring begins, early intervention is crucial.

We found the recommendation to continue dressing of the incision with skin tape during this phase in a review on surgical scar prevention and management [17] and therefore started with application of kinesiotapes as prophylaxis of hypertrophic scar and keloid formation after V1 (after 4–6 weeks post-surgery). During this early remodeling phase, extracellular matrix as an immature healing component is remodeled and type III collagen ultimately matures to type I collagen [10].

Pressure therapy may prevent scar elevation [30] and is advised soon after clinical wound healing. One advantage of kinesiotaping over pressure therapy may be superior patient compliance, as pressure therapy exceeding 24 mmHg for more than 30 min daily as recommended [17] is perceived as physically uncomfortable.

Despite pressure treatment ranking among the most common treatments, pressure garments may not be able to exert adequate pressure due to the complexity of the human body [31]. This is also the case for the scar after gluteal DFG harvesting: The location is under movement and mechanical contact of different layers of clothing. We thus consider it an advantage of kinesiotaping of the gluteal location that the tape effectively mobilizes the hypertrophic skin by consistently moving it against subcutaneous collagenous tissue.

Kinesiotaping is a non-invasive prophylactic as well as therapeutic procedure to improve scar formation with minor adverse effects. In this study, no hypersensitivity reactions or complications due to taping were reported. Application of kinesiotape is easier for the patient than application of silicone (which can be considered among first-line treatments of hypertrophic scars). Kinesiotaping is fitted over the scar without tension and can be left in place for 2–3 weeks, while silicone sheets must be worn over the scar area for 12–24 h daily for 2–3 months or be applied multiple times per day as gel [11].

Inference from this study is limited in different ways: First, enucleation is not a frequent intervention with globe-preserving therapy being possible in many cases and surgery without harvesting of a DFG is possible, such that respective numbers of cases and controls were rather limited despite the 18-month recruitment-period of this prospective, randomized study at a large university center. Furthermore, despite photodocumentation excluding the perianal region, two patients refused participation in the study for reasons of modesty and two patients were incapacitated adults.

Second, patient compliance regarding the application of the tape was assumed as explained within the form of written informed consent but could not be controlled.

Third, scarring is not terminated at 17–19 weeks following surgery, but the scar will mature as the remodeling phase continues. Yet, good results could already be achieved within this limited timeframe and in hope of best patient compliance and associated quality of life, we tried to integrate the scar therapy within routine follow-up visits at our institution. Kinesiotaping could prevent hypertrophic scarring or keloids at an early stage. Long-term follow-up and comparison are warranted.

No functionally impairing or painful scarring occurred in the case or control group proving that in addition to the advantages of an autologous implant (low rate of conjunctival extrusion and postenucleation syndrome, good functional and esthetic long-term outcome, extremely rare rejection rate), DFG harvesting is not a functional disadvantage in patient treatment over enucleation and supply with an alloplastic implant. Our preliminary results show good reduction of color, scar length and prominence already at 4–5 months post-surgery. Kinesiotaping is a cost-efficient tool that is easily applicable and may aid in preventing elevated scarring after gluteal DFG harvesting. This should be validated in further studies with larger patient collectives and longer follow-up.

## Data Availability

The blinded datasets used and analyzed during the current study are available from the corresponding author upon reasonable request.
